# Identification of the alpha linolenic acid metabolism-related signature associated with prognosis and the immune microenvironment in nasopharyngeal carcinoma

**DOI:** 10.3389/fendo.2022.968984

**Published:** 2022-08-05

**Authors:** Zhijie Fang, Hua Huang, Liyu Wang, Zhiqiang Lin

**Affiliations:** ^1^ Department of Otolaryngology, The Affiliated Suzhou Hospital of Nanjing Medical University, Suzhou Municipal Hospital, Gusu School, Nanjing Medical University, Suzhou, China; ^2^ The Affiliated Suzhou Hospital of Nanjing Medical University, Suzhou Municipal Hospital, Gusu School, Nanjing Medical University, Suzhou, China

**Keywords:** nasopharyngeal carcinoma, metabolism, immune, immunotherapy, prognosis

## Abstract

**Background:**

Tumor metabolism is important for cancer progression. Nevertheless, the role of the metabolism pathway and related molecules in nasopharyngeal carcinoma (NPC) is limited.

**Methods:**

Open-accessed data was downloaded from The Cancer Genome Atlas database. All the analysis was performed using the R software and the package in R environments.

**Results:**

In our study, we firstly explored the role of 21 metabolism-related pathways in NPC patients. We found that the steroid biosynthesis and biosynthesis of unsaturated fatty acids were risk factors, while the alpha linolenic acid metabolism was a protective factor. Then, the alpha linolenic acid metabolism aroused our interest. A total of 128 differentially expressed genes (DEGs) were identified, including 71 downregulated and 57 upregulated genes identified between high and low alpha linolenic acid metabolism level. Based on these DEGs, we constructed a prognosis model including DEFB4B, FOXL2NB, MDGA2, RTL1, SLURP2, TMEM151B and TSPAN19, which showed great prediction efficiency in both training and validation cohorts. Clinical correlation analysis showed that high-risk patients might have worse clinical pathology parameters. Pathway enrichment analysis showed that riskscore was positively correlated with angiogenesis, DNA repair, G2/M checkpoints, IL6/JAK/STAT3 signaling, KRAS signaling up, WNT beta-catenin signaling, PI3K/AKT/mTOR signaling, yet positively correlated with inflammatory response, xenobiotic metabolism, TNF-α signaling *via* NFKB and interferon-gamma response. Immune infiltration analysis showed that the riskscore was positively correlated with the M2 and M0 macrophages, but negatively correlated with neutrophils, plasma cells, follicular helper T cells and resting dendritic cells Moreover, we found that the low-risk patients might be more sensitive to immunotherapy and lapatinib.

**Conclusions:**

In all, our study identified the genes associated with alpha linolenic acid metabolism and constructed an effective prognosis model which could robustly predict NPC patients prognosis.

## Introduction

Nasopharyngeal carcinoma (NPC) develops from the nasopharyngeal epithelium cells and is a type of head and neck cancer, which is common in south China and Southeast Asia (1). Globally, NPC can lead to approximately 80,000 new cases and 50,000 deaths each year (1). As a complex and multifactorial disease, NPC may be triggered by a variety of factors, including viral, genetic and environmental factors (2). Most NPC patients might have cervical nodal metastases when the first diagnosis. The standard therapy consisting of concurrent chemo-radiotherapy with cisplatin-based regimens could cure the vast majority of patients (3). However, there are still challenges in preventing disease recurrence, treating patients with refractory or metastatic NPC, and long-term toxicity management.

Tumor metabolism is important for the proliferation and expansion of cancer cells (4). The specific metabolic states could contribute to the biological process closely related to tumor growth (5). Recently, growing attention has been paid to the metabolism status of cancer (6). Also, targeting the hub molecules involved in tumor metabolism is becoming an emerging therapeutic option for cancer (7). For example, Kodama et al. indicated that the shift in glutamine nitrogen metabolism contributes to the malignant progression of cancer, especially in neuroendocrine cancer including small cell lung cancer (8). Tang et al. found that c-MYC-directed NRF2 could drive the head and neck cancer progression through glucose-6-phosphate dehydrogenase and transketolase activation (9). Li et al. revealed that TRIM47 could accelerate aerobic glycolysis and tumor progression by regulating ubiquitination of FBP1 in pancreatic cancer (10). Meanwhile, Li et al. found that lncRNA Ftx could facilitate aerobic glycolysis and tumor progression through the PPARγ pathway in hepatocellular carcinoma (11). In NPC, Zheng et al. found that the lncRNA TINCR-mediated regulation of acetyl-CoA metabolism could promote NPC progression and chemoresistance through the TINCR-ACLY-PADI1-MAPK-MMP2/9 axis (12). Hong et al. found that the circular RNA CRIM1 functions as a ceRNA to promote NPC metastasis and docetaxel chemoresistance through upregulating FOXQ1 (13). Therefore, exploration of the metabolism status and related molecules in NPC might be helpful for NPC treatment.

Nowadays, the advancement of bioinformatics analysis can effectively help researchers find novel molecules involved in disease development (14). In our study, through comprehensive bioinformatic analysis, we explored the metabolism pathway in NPC. The alpha linolenic acid metabolism aroused our interest and was selected for further analysis. Next, a total of 128 DEGs were identified as alpha linolenic acid metabolism-related genes through differentially expressed genes (DEGs) analysis. Based on these DEGs, we constructed a prognosis model based on DEFB4B, FOXL2NB, MDGA2, RTL1, SLURP2, TMEM151B and TSPAN19, which showed great prediction efficiency in both training and validation cohorts. Clinical correlation analysis showed that high-risk patients might have worse clinical pathology parameters. Pathway enrichment analysis was then performed to explore the underlying biological differences between high- and low-risk patients. Immune infiltration analysis showed that the riskscore was positively correlated with the M2 and M0 macrophages, but negatively correlated with neutrophils, plasma cells, follicular helper T cells and resting dendritic cells. Moreover, we found that the low-risk patients might be more sensitive to immunotherapy.

## Methods

### Data acquisition

The expression profile and clinical data of patients used in this study were obtained from The Cancer Genome Atlas (TCGA) databases (TCGA-HNSC project). Detailed, the expression profile was firstly downloaded with the “STAR-Counts” form and then collated as the “TPM” form using the R code, The reference genome file Homo_sapiens.GRCh38.106.chr.gtf obtained from the Ensembl website (http://asia.ensembl.org/index.html) was used for gene annotation. Clinical information files were “bcr xml” form and collated using the Perl code. A standardized process was performed before the analysis, including probe annotation, missing value completion, data normalization. The baseline information of patients in our analysis was shown in **Table 1**.

**Table 1:** The baseline data of patients.

Features		Numbers	Proportion
Age	<=60	260	49.2%
	>60	267	50.6%
	Unknown	1	0.2%
Gender	Female	142	26.9%
	Male	386	73.1%
Grade	G1	63	11.9%
	G2	311	58.9%
	G3	125	23.7%
	G4	7	1.3%
	Unknown	22	4.2%
Stage	Stage I	27	5.1%
	Stage II	74	14.0%
	Stage III	82	15.5%
	Stage IV	270	51.1%
	Unknown	75	14.2%
T classification	T0	1	0.2%
	T1	49	9.3%
	T2	140	26.5%
	T3	101	19.1%
	T4	175	33.1%
	Unknown	62	11.7%
M classification	M0	191	36.2%
	M1	1	0.2%
	Unknown	336	63.6%
N classification	N0	180	34.1%
	N1	68	12.9%
	N2	172	32.6%
	N3	8	1.5%
	Unknown	100	18.9%

### Metabolism status quantification

The quantification of metabolism status was performed using the single sample gene set enrichment analysis (ssGSEA) algorithm (15). The reference pathway file is shown in **Table S1**. Heatmap was used to show the quantification result.

### DEGs analysis and protein-protein interaction (PPI) network

DEGs analysis was performed based on the limma package with the threshold of |log FC| > 2 and P.value < 0.05 (16). The STRING database was used to construct the PPI network (17). Detailed, the Organism was “Homo sapiens”. The interaction was “medium confidence”. Cytoscape software v3.7.2 was used for the PPI network visualization. Cytohubba plug-in in Cytoscape was used to identify the hub nodes according to the calculated importance. ClueGO was used to perform pathway enrichment analysis of the selected nodes, which is a plug-in in cytoscape software (18). ClueGO can provide an intuitive representation of the gene oncology (GO) and Kyoto Encyclopedia of Genes and Genomes (KEGG) analysis of the selected nodes.

### Prognosis-model construction and evaluation

Patients were randomly divided into the training group and validation group with a 1:1 ratio. Based on the identified genes, univariate Cox regression analysis was first performed to identify the prognosis-related genes. Next, the LASSO regression and multivariate Cox regression analysis were performed for model construction with the formula of “riskscore = Gene A * coef A + Gene B * coef B + Gene C * coef C + … + Gene N * coef N” (19, 20). LASSO regression could effectively avoid overfitting, which is a regularization method that is equipped with in-built feature selection (19). Each patient was assigned with a riskscore and patients were divided into high- and low-risk group according to the median riskscore. Kaplan-Meier survival and receiver operating characteristic (ROC) curves were used for the evaluation of the prognosis model. Further, univariate analysis and multivariate analysis were performed to assess the independence of our model. A nomogram plot was established by combining the riskscore and clinical features using the rms package. Calibration curves were used to evaluate the fitting degree of nomogram predicted survival and actual survival.

### Pathway enrichment and genomic instability analysis

Pathway enrichment analysis was conducted using the Gene set variation analysis (GSVA) and Gene set enrichment analysis (GSEA) algorithms (15, 21). GSVA analysis was performed using the GSVA package in R environments. GSEA analysis was performed using the clusterProfiler in R environments. Tumor mutational burden (TMB) and microsatellite instability (MSI) score were obtained from the TCGA database. Tumor stemness was calculated one-class logistic regression machine learning (OCLR) machine-learning algorithm (22). The mutation information was obtained from the cBioPortal website (https://www.cbioportal.org/).

### Immune-related and drug sensitivity analysis

Immune infiltration was performed based on the CIBERSORT algorithm, which could quantify the relative abundance of 21 immune cell types (23). The immunotherapy sensitivity was analyzed based on the Tumor Immune Dysfunction and Exclusion (TIDE) algorithm (24). Drug sensitivity analysis was performed based on the Genomics of Drug Sensitivity in Cancer (GDSC) (25).

### Statistical analysis

All the statistical analysis were performed in R software v3.7.2. All the P.values were two-sided and less than 0.05 was considered statistically significant. Student T-test was used for the continuous variables normally distributed. Mann–Whitney U test was used for the continuous variables normally distributed.

## Results

### Exploration of the metabolic pathways in cancer patients

**Figure 1:**
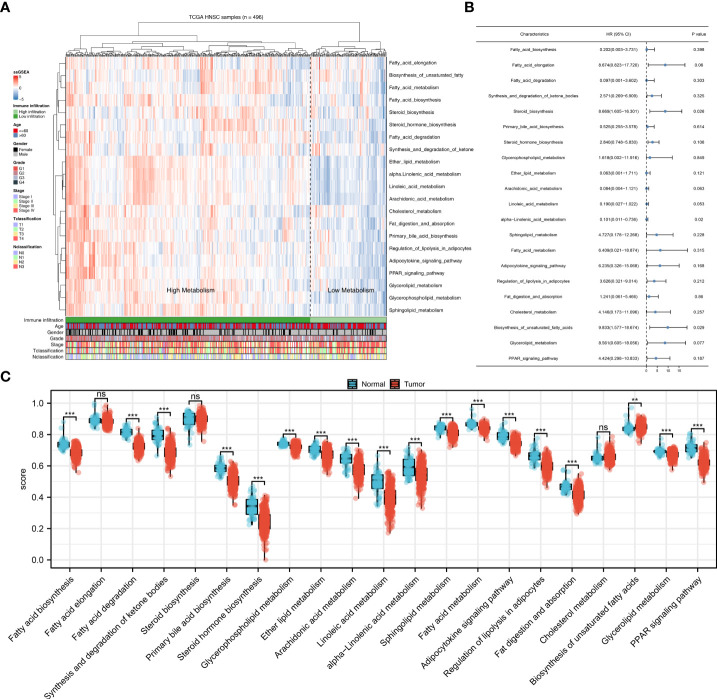
Quantification of the metabolism pathways. **(A)** ssGSEA algorithm was used to quantify the 21 metabolism pathways; **(B)** Univariate Cox analysis was used to identify the prognosis-related metabolism pathways; **(C)** The 21 metabolism pathways difference in normal and tumor tissue. **P < 0.01; ***P < 0.001; ns, P > 0.05.

The flow chart of the whole study was shown in **Figure S1**. Based on the ssGSEA algorithm, 21 metabolism-related pathways were quantified, which was shown in **Figure 1A**. Then, univariate Cox analysis was performed to screen the prognosis-related metabolism pathways. The result showed that the steroid biosynthesis and biosynthesis of unsaturated fatty acids were risk factors, while the alpha linolenic acid metabolism was a protective factor (**Figure 1B**). Further, we explore the difference between these metabolic pathways in tumor and normal tissue. The result showed most metabolic pathways had a significant difference between the tumor and normal tissue, indicating that NPC patients might have a different metabolic status compared with the normal tissue (**Figure 1C**). Considering that alpha linolenic acid metabolism was downregulated in tumor tissue and also a protective factor, it aroused our interest and was selected for further analysis.

### Identification of genes associated with alpha linolenic acid metabolism

**Figure 2:**
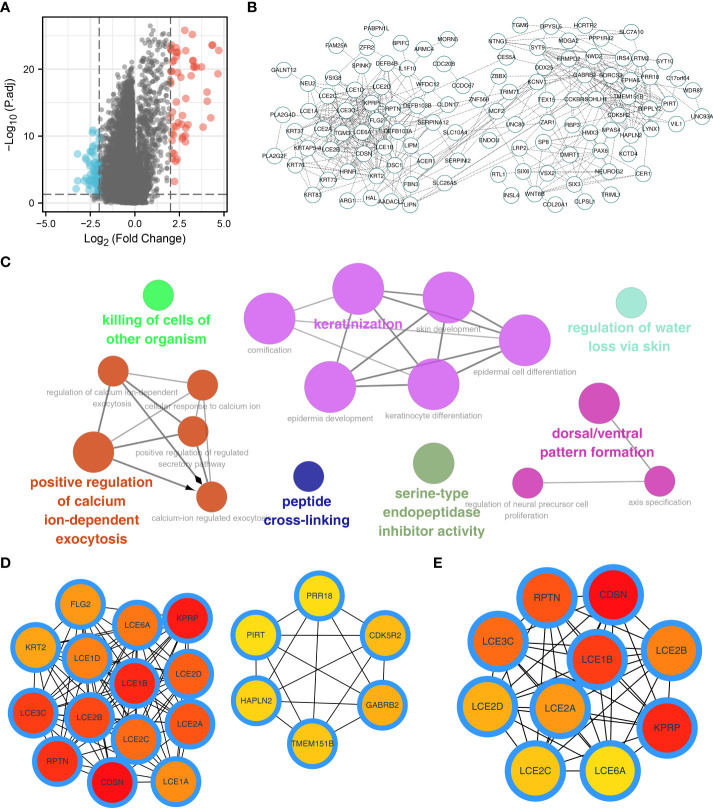
Identification of the DEGs of alpha linolenic acid metabolism. **(A)** Limma package was used to identify the DEGs between high and low alpha linolenic acid metabolism**; (B)** PPI network of the DEGs; **(C)** ClueGO analysis of the DEGs; **(D–E)** Top 20 and 10 important nodes of the DEGs.

Next, we performed DEGs analysis between patients with high and low alpha linolenic acid metabolism status with the threshold of |log FC| > 2 and P.value < 0.05. A total of 128 DEGs were identified, including 71 downregulated and 57 upregulated genes (**Figure 2A**). Based on all the DEGs, the PPI network was constructed (**Figure 2B**). ClueGO plug-in showed that these nodes were significantly enriched in the process of pathway regulation of calcium ion-dependent exocytosis, killing of cells of other organism, keratinization, peptide cross-linking, serine-type endopeptidase inhibitor activity, regulation of water loss *via* skin and dorsal/ventral pattern formation (**Figure 2C**). The top 20 important nodes were shown in **Figure 2D**. The top ten important nodes were shown in **Figure 2E**, including RPTN, CDSN, LCE3C, LCE1B, LCE2B, LCE2D, LCE2A, KPRP, LCE2C and LCE6A.

### Model construction and validation

**Figure 3:**
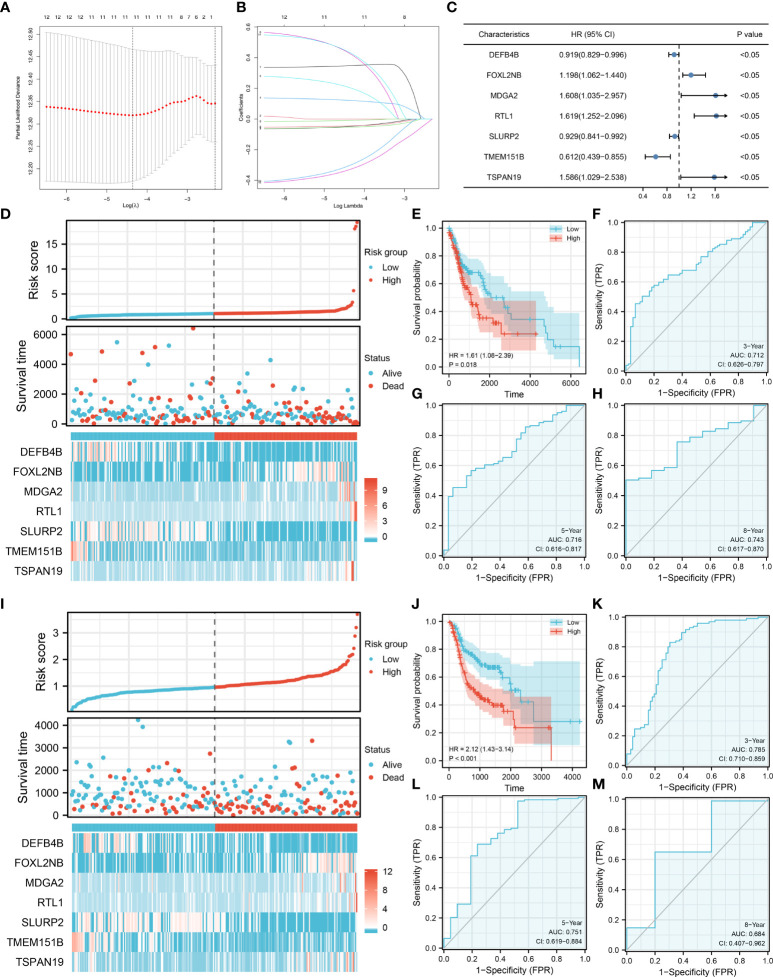
Model construction and validation. **(A–B)** LASSO regression analysis was performed for dimensionality reduction; **(C)** Multivariate Cox analysis was performed for prognosis model construction, including DEFB4B, FOXL2NB, MDGA2, RTL1, SLURP2, TMEM151B and TSPAN19; **(D)** The overview of prognosis model in training cohort; **(E–H)** Kaplan-Meier survival and ROC curves for prognosis model evaluation in the training cohort; **(I)** The overview of prognosis model in validation cohort; **(J–M)** Kaplan-Meier survival and ROC curves for prognosis model evaluation in the validation cohort.

Based on the DEGs identified, univariate Cox regression analysis was performed to identify prognosis-related genes (**Table 2**). LASSO regression analysis was performed for dimensionality reduction (**Figures 3A, B**). Multivariate Cox regression analysis identified seven genes for prognosis model construction, including DEFB4B, FOXL2NB, MDGA2, RTL1, SLURP2, TMEM151B and TSPAN19 (**Figure 3C**). The riskscore was calculated with the formula of “Riskscore = DEFB4B * -0.084 + FOXL2NB * 0.181 + MDGA2 * 0.475 + RTL1 * 0.483 + SLURP2 * -0.073 + TMEM151B * -0.489 + TSPAN19 * 0.461”. According to the median riskscore, patients were divided into high- and low-risk groups. In the training cohort, a higher proportion of dead cases was observed (**Figure 3D**). Kaplan-Meier survival curve showed that the high-risk patients might have a worse prognosis (**Figure 3E**, HR = 1.61, P = 0.018). ROC curves showed that our model had a great prediction efficiency in patients prognosis (**Figures 3F–H**, 3-year AUC = 0.712, 5-year AUC = 0.716, 8-year AUC = 0.743). The same trend was also noticed in the validation group (**Figure 3I**). Kaplan-Meier survival curve indicated that in the validation group, the high-risk patient might have a shorter overall survival (OS) (**Figure 3J**, HR = 2.12, P < 0.001). ROC curves also indicated that the model had a good prediction efficiency in the validation group (**Figures 3K–M**, 3-year AUC = 0.785, 5-year AUC = 0.751, 8-year AUC = 0.684).

**Table 2:** Prognosis-related genes identified by univariate Cox regression analysis.

**id**	**HR**	**HR.95L**	**HR.95H**	**pvalue**
RTL1	1.728	1.362	2.192	0.000
ZFR2	0.736	0.610	0.887	0.001
FOXL2NB	1.260	1.068	1.487	0.006
ENDOU	0.900	0.826	0.981	0.017
DYNAP	0.864	0.762	0.979	0.022
SLURP2	0.899	0.820	0.985	0.022
SPINK7	0.940	0.891	0.992	0.024
TSPAN19	1.630	1.061	2.503	0.026
MDGA2	1.906	1.072	3.389	0.028
TMEM151B	0.725	0.544	0.966	0.028
GFY	1.280	1.026	1.598	0.029
DEFB4B	0.899	0.813	0.994	0.038

### Clinical correlation analysis

**Figure 4:**
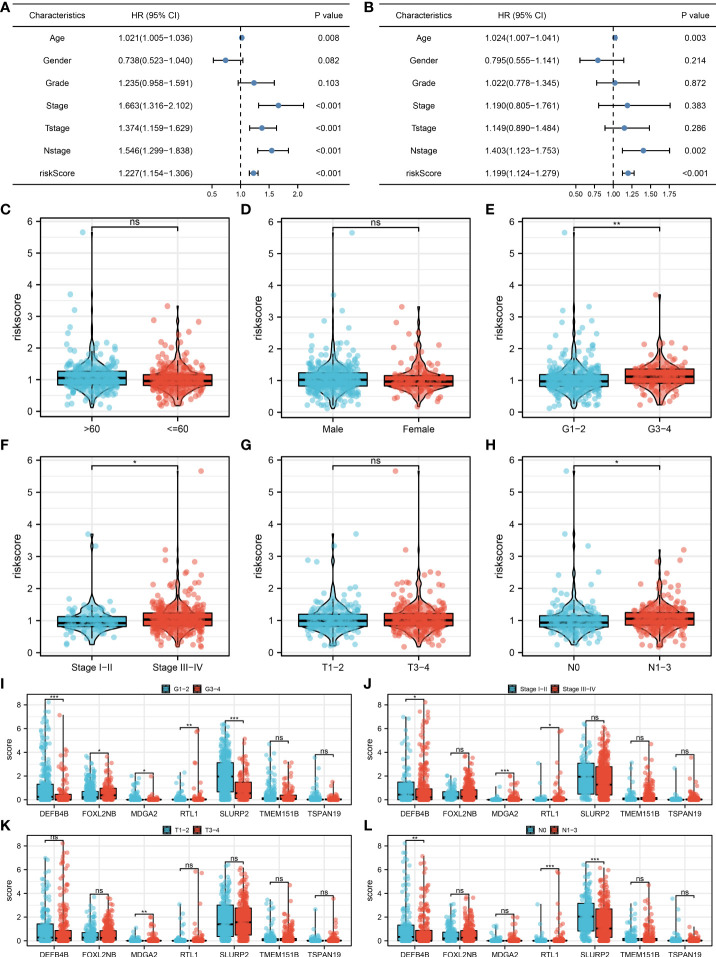
Clinical correlation analysis. **(A)** Univariate analysis was performed to evaluate the independence of our model; **(B)** Multivariate analysis was performed to evaluate the independence of our model; **(C–H)** The riskscore difference between patients with different clinical features; **(I–L)** The expression difference of model genes in patients with different clinical features. *P < 0.05; **P < 0.01; ***P < 0.001; ns. P > 0.05.

Univariate and multivariate analysis were performed to evaluate the independence of our model. The result showed that the riskscore is a risk factor independent of other clinical features (**Figures 4A, B**, univariate analysis, HR = 1.227, P < 0.001; multivariate analysis, HR = 1.199, P < 0.001). Clinical correlation analysis showed that the patients with more progressive grade, N classification and clinical stage might have a higher riskscore, yet no significant difference was observed in age, gender and T classification (**Figures 4C–H**). For the model genes, we found that the G3-4 patients had a higher FOXL2NB, MDGA2, RTL1 expression, but a lower DEFB4B and SLURP2 expression compared to the G1-2 patients (**Figure 4I**); the stage III-IV patients had a higher MDGA2 and RTL1 expression, but a lower DEFB4B expression compared to the stage I-II patients (**Figure 4J**); the T3-4 patients had a higher MDGA2 expression compared to the T1-2 patients (**Figure 4K**); the N1-3 patients had a higher RTL1 expression, but a lower DEFB4B and SLURP2 expression compared to the N0 patients (**Figure 4L**). Moreover, a nomogram plot was constructed by combining the riskscore and clinical features (**Figure S2A**). Calibration curves indicated a great fitting degree of nomogram predicted survival and actual survival (**Figure S2B**).

### Pathway enrichment analysis

**Figure 5:**
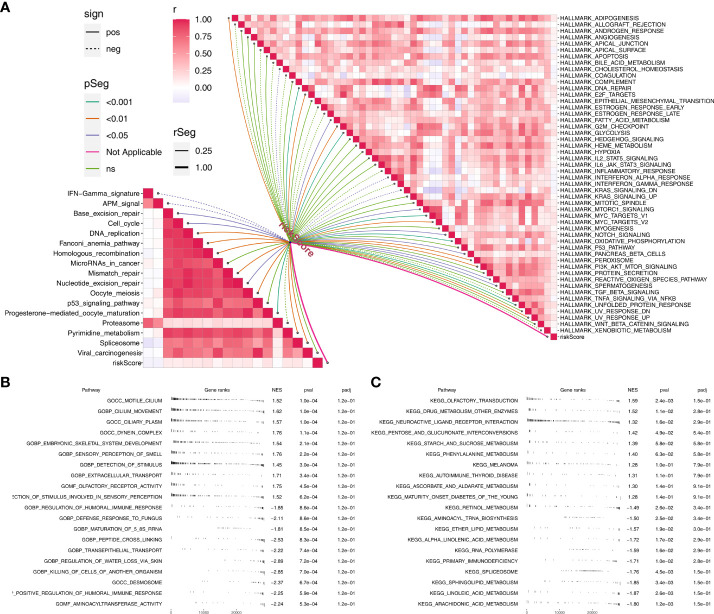
Pathway enrichment analysis. **(A)** The correlation of riskscore with immune and hallmark pathways; **(B)** GSEA analysis based on GO terms; **C:** GSEA analysis based on KEGG terms.

Based on the Hallmark gene set, pathway enrichment analysis showed that the riskscore was positively correlated with angiogenesis, DNA repair, G2/M checkpoints, IL6/JAK/STAT3 signaling, KRAS signaling up, WNT beta-catenin signaling, PI3K/AKT/mTOR signaling, yet positively correlated with inflammatory response, xenobiotic metabolism, TNF-α signaling *via* NFKB and interferon-gamma response (**Figure 5A**). For immune-related pathways, the riskscore was positively correlated with base excision repair, cell cycle, DNA replication, fanconi anemia pathway, homologous recombination, micRNAs in cancer, mismatch repair, nucleotide excision repair, oocyte meiosis, progesterone mediated oocyte maturation, pyrimidine metabolism, spliceosome and viral carcinogenesis, but positively correlated with IFN-gamma signature, APM signal and proteasome (**Figure 5A**). Based on the GSEA analysis of GO, we found that the terms of motile cilium, cilium movement, ciliary plasm, dynein complex, embryonic skeletal system development were significantly enriched in high-risk group (**Figure 5B**). Also, for the KEGG, the terms of olfactory transduction, drug metabolism other enzymes, neuroactive ligand receptor interconversions and starch and sucrose metabolism were remarkably enriched in high-risk group (**Figure 5C**).

### Genomic instability analysis

**Figure 6:**
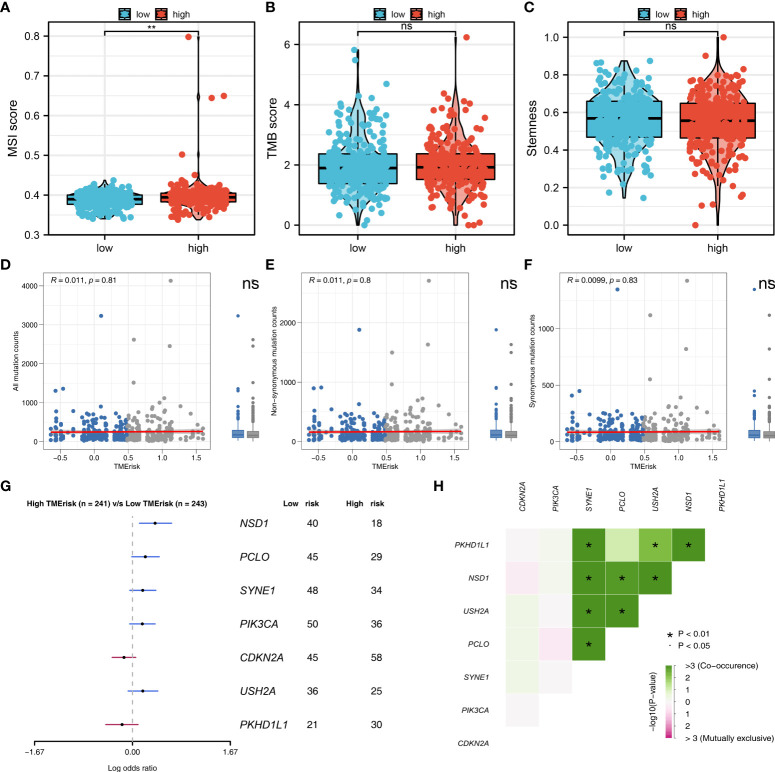
Genomic instability analysis. **(A–C)** The MSI, TMB and tumor stemness difference in high- and low-risk group; **(D–F)** The all mutation counts, non-synonymous mutation counts and synonymous mutation counts difference between high- and low-risk patients; **(G)** High-risk patients might be more inclined to have NSD1, PCLO, SYNE1, PIK3CA and USH2A mutated, but not CDKN2A and PKDH1L1; **(H)** The co-mutated correlation of these genes. **P < 0.01.

We further explored the genomic differences between high- and low-risk patients. The result showed that the high-risk patients might have a higher MSI score compared to the low-risk patients (**Figure 6A**). However, no remarkable difference was observed in TMB score and tumor stemness between high- and low-risk patients (**Figures 6B, C**). Meanwhile, the difference were not significant in all mutation counts, non-synonymous mutation counts and synonymous mutation counts between high- and low-risk patients (**Figures 6D–F**). Moreover, we found that the high-risk patients might be more inclined to have NSD1, PCLO, SYNE1, PIK3CA and USH2A mutated, but not CDKN2A and PKDH1L1 (**Figure 6G**). The co-mutated correlation was shown in **Figure 6H**.

### Immune-related and drug sensitivity analysis

**Figure 7:**
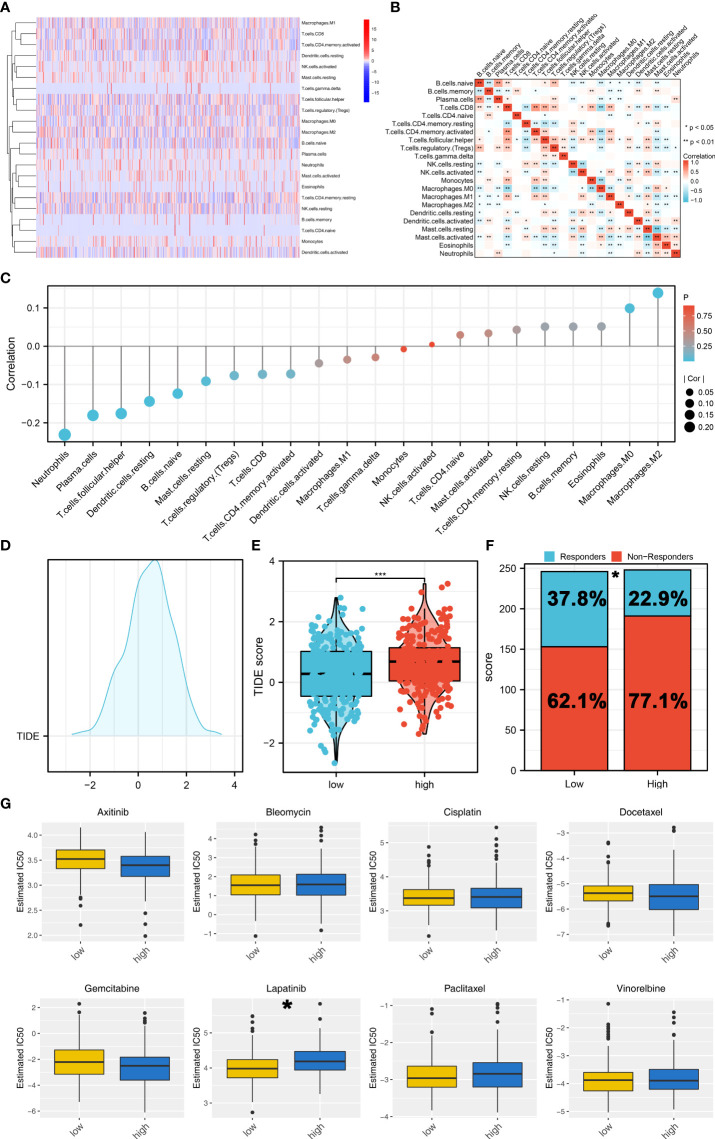
Immune related analysis. **(A)** CIBERSORT algorithm was used to quantify the 21 immune cells in tumor microenvironment; **(B)** The co-expression analysis of these immune cells; **(C)** The correlation of the riskscore and immune cells; **(D)** TIDE analysis was performed to assess the sensibility of immunotherapy; **(E)** The low-risk patients might have a lower TIDE score; **(F)** A higher percentage of immunotherapy responders was observed in low-risk patients; **(G)** Drug sensitivity analysis between high and low risk groups. *P < 0.05; *** P < 0.001.

Tumor immune microenvironment could affect cancer progression. Therefore, we try to explore the immune infiltration differences between high- and low-risk patients. CIBERSORT algorithm was used to quantify the immune cell infiltration in tumor tissue (**Figure 7A**). The co-expression relationship of these immune cells was shown in **Figure 7B**. Moreover, we found that the riskscore was positively correlated with the M2 and M0 macrophages, but negatively correlated with neutrophils, plasma cells, follicular helper T cells and resting dendritic cells (**Figure 7C**). TIDE analysis was then performed to explore the immunotherapy sensitivity, of which the patients with TIDE <0 were defined as immunotherapy responders and > 0 were defined as immunotherapy non-responders (**Figure 7D**). Moreover, we found that the low-risk patients might have a lower TIDE score than that in high-risk group (**Figure 7E**). A higher percentage of immunotherapy responders was observed in low-risk patients, indicating that low-risk patients might be more sensitive to immunotherapy (**Figure 7F**). Drug sensitivity analysis showed that the low risk patients might be more sensitive to lapatinib, while no significant difference was observed in axitinib, bleomycin, cisplatin, docetaxel, gemcitabine, paclitaxel and vinorelbine (**Figure 7G**).

## Discussion

NPC is a common head and neck malignancy, which is associated with viral, genetic and environmental factors (26). Though radiotherapy can bring significant therapeutic benefits for early-stage NPC, however, for these advanced patients, the prognosis is still unsatisfactory (26).

Recently, tumor metabolism is advancing rapidly. Also, the association between tumor metabolism and tumor microenvironment received increasing attention (27). Even if in the nutrient limitation conditions, the metabolic reprogramming in cancer can still supported the proliferation and growth of tumor cells (27). Some biological molecules has been found associated with cancer progression through metabolic manners (28). Nie et al. found that O-GlcNAcylation of PGK1 coordinates glycolysis and TCA cycle to promote tumor growth in colon cancer (29). Cheng et al. indicated that the TRIM21 and PHLDA3 negatively regulate the crosstalk between the PI3K/AKT pathway and pentose phosphate pathway metabolism, which might be an underlying therapeutic target for cancers with PTEN loss or PI3K/AKT activation (30).

In our study, we firstly explored the role 21 metabolism-related pathways in NPC patients. We found that the steroid biosynthesis and biosynthesis of unsaturated fatty acids were risk factors, while the alpha linolenic acid metabolism was a protective factor. Then, the alpha linolenic acid metabolism aroused our interest. A total of 128 DEGs were identified, including 71 downregulated and 57 upregulated genes identified between high and low alpha linolenic acid metabolism level. Based on these DEGs, we constructed a prognosis model including DEFB4B, FOXL2NB, MDGA2, RTL1, SLURP2, TMEM151B and TSPAN19, which showed great prediction efficiency in both training and validation cohorts. Clinical correlation analysis showed that high-risk patients might have worse clinical pathology parameters. Pathway enrichment analysis was then performed to explore the underlying biological differences between high- and low-risk patients. Immune infiltration analysis showed that the riskscore was positively correlated with the M2 and M0 macrophages, but negatively correlated with neutrophils, plasma cells, follicular helper T cells and resting dendritic cells Moreover, we found that the low-risk patients might be more sensitive to immunotherapy. Drug sensitivity analysis showed that the low risk patients might be more sensitive to lapatinib

Our study established a prognosis model consisting of seven linolenic acid metabolism related genes DEFB4B, FOXL2NB, MDGA2, RTL1, SLURP2, TMEM151B and TSPAN19. In HNSC, some prognosis models including specific molecules have been established. For example, Zhu et al. developed a prediction model for radiotherapy response among HNSC patients based on the tumor immune microenvironment and hypoxia signature (31). To the best of our knowledge, this is the first study focused on the role of alpha linolenic acid metabolism and its related genes in NPC. In addition to predicting the prognosis of NPC patients, our model can also indicate the immunotherapy sensitivity of patients, which might have underlying clinical application potential. In the clinical, NPC tissues of patients can be obtained through postoperative or other endoscopic operations. Then, detection of the expression level of model genes through absolute quantification tool could predict the prognosis and therapy option.

Alpha linolenic acid metabolism is an extremely important metabolic pathway in human (32). Meanwhile, the classic metabolism pathway could also significantly affect the biological behavior of cancer (33). Wang et al. revealed that alpha-linolenic acid could suppress the migration of human triple-negative breast cancer cells by attenuating Twist1 expression and suppressing Twist1-mediated epithelial-mesenchymal transition (EMT) (34). Li et al. found that in the mouse model, dietary supplementation of α-linolenic acid induced conversion of n-3 LCPUFAs and reduced prostate cancer growth (35). However, there is a little study focused on the role of alpha linolenic acid metabolism in NPC. In our study, we found that alpha-linolenic acid metabolism is a protective factor of NPC, which might provide direction for future studies.

Pathway enrichment analysis showed that riskscore was positively correlated with angiogenesis, DNA repair, G2/M checkpoints, IL6/JAK/STAT3 signaling, KRAS signaling up, WNT beta-catenin signaling, PI3K/AKT/mTOR signaling. As is well known, angiogenesis is one of the hallmarks of cancer (36). In NPC, the hyperactivation of angiogenesis can facilitate cancer metastasis (37). G2/M checkpoint is a key rate-limiting step of the cell cycle and the abnormal of it could significantly influence the malignant biological behavior of cancer cells (38). PI3K/AKT/mTOR signaling pathway is a classic oncogenic pathway. In NPC, Liu et al. indicated that APLNR is involved in ATRA-induced growth inhibition of NPC and might suppress EMT through PI3K-AKT-mTOR signaling (39). The results indicated that high-risk patients might the a higher activation level of these pathways, therefore leading to a poor prognosis. Meanwhile, we found that the high risk patients might have higher MSI score. A higher genome instability level in cancer might lead to poor prognosis, tumor heterogeneity, and resistance to therapy, which was also partly responsible for the poor prognosis of high risk patients (40).

Immune infiltration analysis showed that riskscore was positively correlated with the M2 macrophages, but negatively correlated with neutrophils, plasma cells, follicular helper T cells and resting dendritic cells. Generally, M2 macrophages are regarded as a cancer-promoting factor in solid tumor (41). Peng et al. found that the microRNA-18a from M2 Macrophages could inhibit TGFBR3, further promoting NPC progression and tumor growth by the TGF-β signaling pathway (42). In addition, Zhang et al. found that EB virus-induced ATR activation could accelerate NPC growth through M2-type macrophage polarization (43). Meanwhile, our results indicated that the low-risk patients were more sensitive to immunotherapy, indicating the underlying potential of our model for individualized treatment.

Some limitations still existed in our study. Firstly, the cases used for our analysis were mainly Western populations. Considering the underlying biological differences in races, it might reduce the stability of our conclusions about other races. Secondly, though NPC is a common pathologic subtype of HNSC, the detailed pathologic information of TCGA-HNSC patients was not provided. Therefore, if the clinical and pathologic data of the enrolled patients were complete, our conclusion would be more credible. Thirdly, the result of our analysis is based on RNA level, but not protein level, which might reduce the reliability of our conclusions.

## Data availability statement

The original contributions presented in the study are included in the article/**Supplementary Material**. Further inquiries can be directed to the corresponding author.

## Author contributions

ZL conceived and designed the whole project and drafted the manuscript. ZF analyzed the data and wrote the manuscript. HH carried out data interpretations and helped data discussion. LW provided specialized expertise and collaboration in data analysis. All authors read and approved the final manuscript.

## Funding

This work was supported by the Science and Technology Development Plan Project of Suzhou (SYSD2020141) and the Medical and Health Technology Innovation Key Technology Project of Suzhou (SKY2021055).

## Conflict of interest

The authors declare that the research was conducted in the absence of any commercial or financial relationships that could be construed as a potential conflict of interest.

## Publisher’s note

All claims expressed in this article are solely those of the authors and do not necessarily represent those of their affiliated organizations, or those of the publisher, the editors and the reviewers. Any product that may be evaluated in this article, or claim that may be made by its manufacturer, is not guaranteed or endorsed by the publisher.
