# Supplementary figures and images for: Identification of the alpha linolenic acid metabolism-related signature associated with prognosis and the immune microenvironment in nasopharyngeal carcinoma
Source: Front Endocrinol (Lausanne). 2022 Aug 5;13:968984. doi: 10.3389/fendo.2022.968984 (PMC9388792; doi:10.3389/fendo.2022.968984)

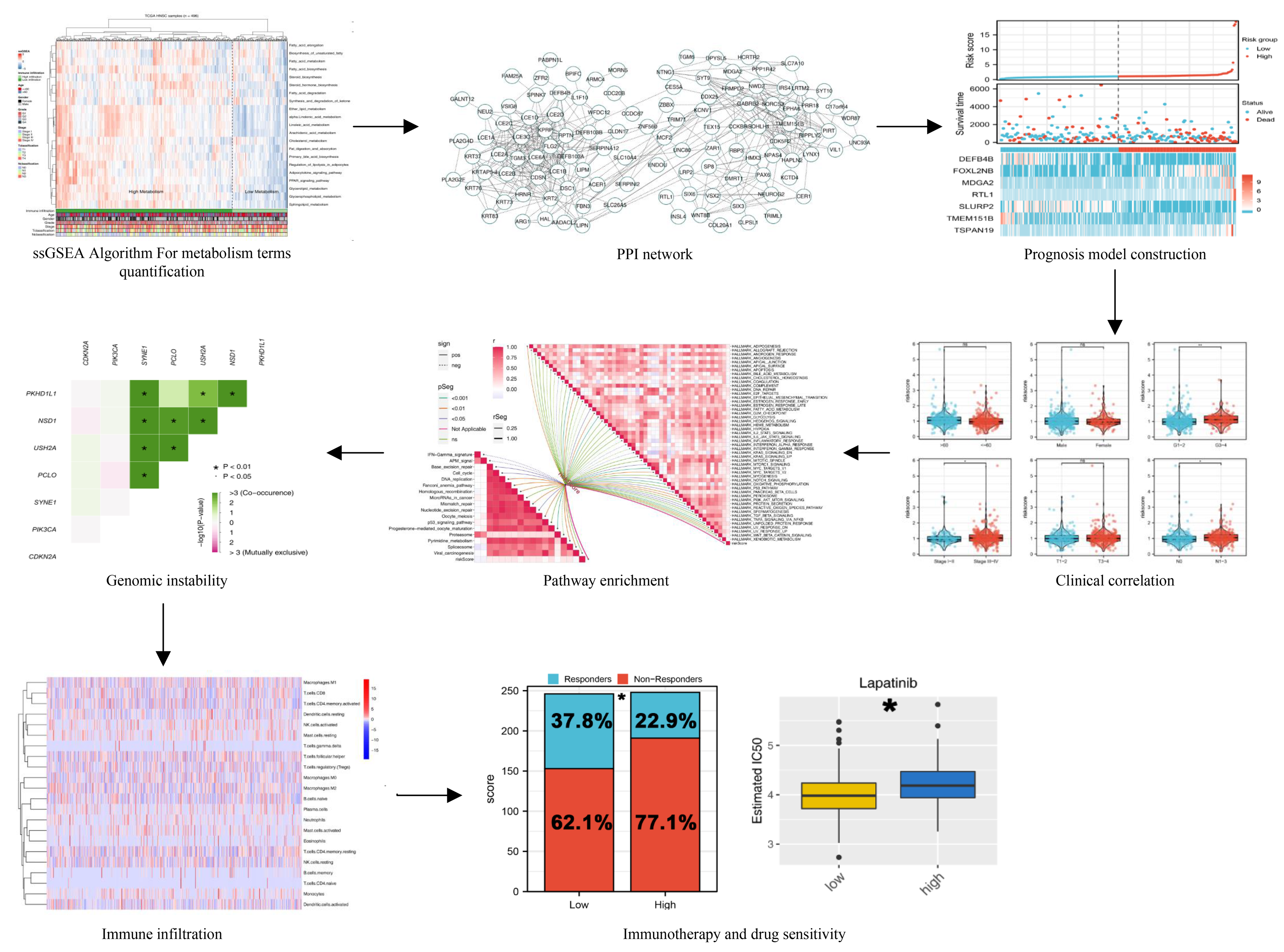

Supplement: Supplementary file 1 [file Image_1.tif]

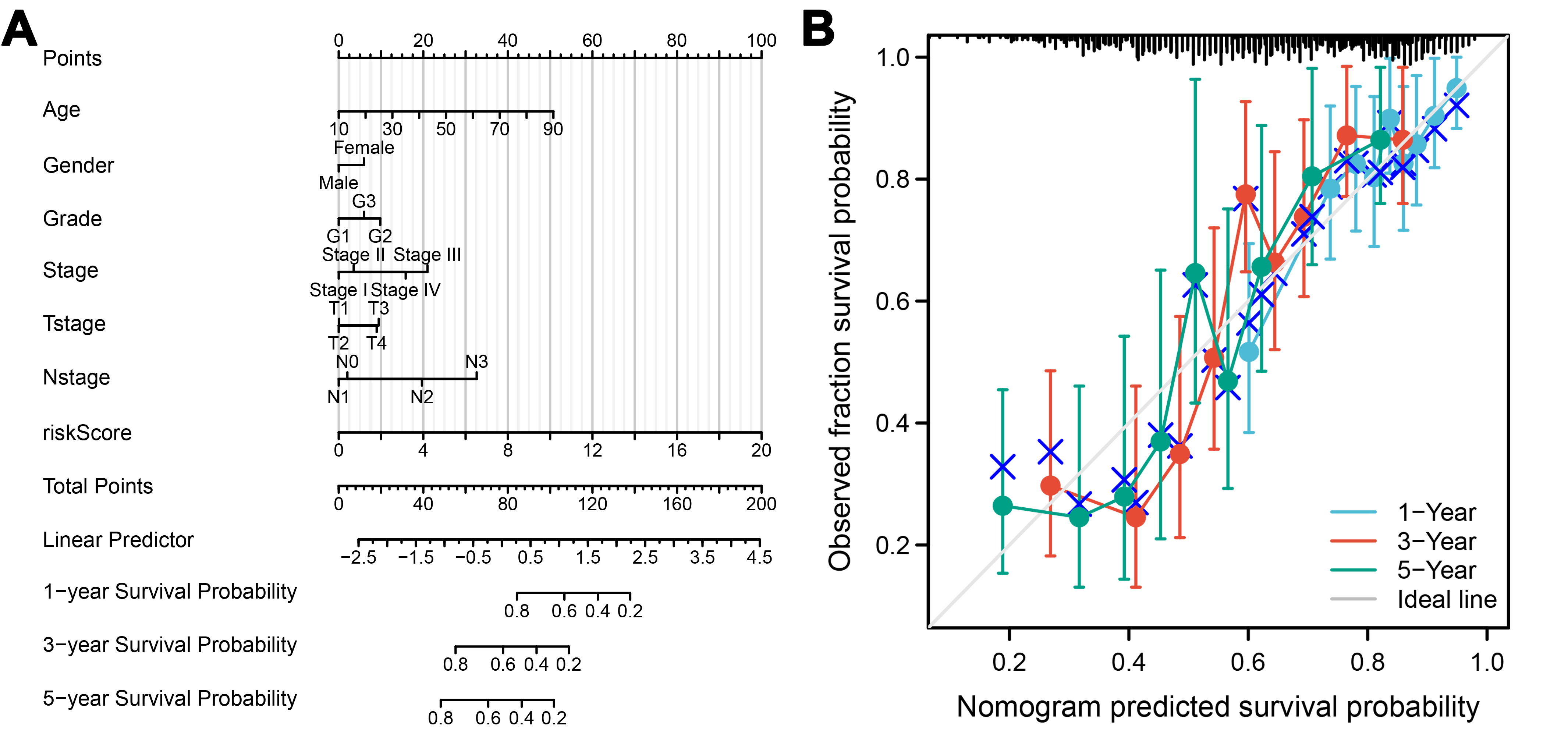

Supplement: Supplementary file 2 [file Image_2.tif]
